# Method combining BAC film and positive staining for the characterization of DNA intermediates by dark-field electron microscopy

**DOI:** 10.1093/biomethods/bpaa012

**Published:** 2020-07-07

**Authors:** Yann Benureau, Eliana Moreira Tavares, Ali-Akbar Muhammad, Sonia Baconnais, Eric Le Cam, Pauline Dupaigne

**Affiliations:** b1 DSB Repair, Replication Stress and Genome Integrity, UMR9019-CNRS ‘Genome Integrity and Cancer’, CNRS, Université Paris-Saclay, Gustave Roussy, F-94805, Villejuif Cedex, France; b2 UMR9019-CNRS, Genome Integrity and Cancer, Equipe labellisée Ligue contre le Cancer, Université Paris-Saclay, Gustave Roussy, F-94805, Villejuif Cedex, France

**Keywords:** transmission electron microscopy, DNA, replication fork, homologous recombination, replication intermediate, recombination intermediate

## Abstract

DNA intermediate structures are formed in all major pathways of DNA metabolism. Transmission electron microscopy (TEM) is a tool of choice to study their choreography and has led to major advances in the understanding of these mechanisms, particularly those of homologous recombination (HR) and replication. In this article, we describe specific TEM procedures dedicated to the structural characterization of DNA intermediates formed during these processes. These particular DNA species contain single-stranded DNA regions and/or branched structures, which require controlling both the DNA molecules spreading and their staining for subsequent visualization using dark-field imaging mode. Combining BAC (benzyl dimethyl alkyl ammonium chloride) film hyperphase with positive staining and dark-field TEM allows characterizing synthetic DNA substrates, joint molecules formed during not only *in vitro* assays mimicking HR, but also *in vivo* DNA intermediates.

## Introduction

DNA metabolism pathways including transcription, replication, repair, and recombination involve DNA transactions with transient conformational states and intermediates structures formation. The fine regulation of these DNA intermediates is not only critical for accurate genome duplication, expression, but also strongly participates in the genome maintenance and plasticity. DNA is constantly threatened by exogenous or endogenous challenges producing damages that can alter its structure and consequently the progression of the replication fork, leading to potential genomic instability. Cells have, therefore, evolved several DNA damage response (DDR) pathways to limit the accumulation of damages, insoluble DNA intermediates, and DNA breakages [1]. Precisely, replication stress is accompanied by alterations in the replication fork structure and the subsequent formation of double- and single-stranded DNA (ds and ssDNA) breaks leading to the activation of the intra-S-phase checkpoint and to the potential recruitment of DNA repair and/or tolerance machinery [2, 3]. In particular, homologous recombination (HR) using the undamaged homologous sister chromatid to repair the damaged DNA is a pivotal pathway involved in these processes [4]. The structural characterization of the DNA intermediates choreography is crucial to better understand these major DDR pathways. 

Conventional transmission electron microscopy (TEM) is a pertinent and powerful tool for the observation of DNA molecules allowing the unique opportunity to characterize DNA conformation and topology, nucleoprotein complexes, but also the architecture of various DNA intermediates. In TEM studies, the spreading of DNA molecules onto the carbon film surface of copper grids is a critical step. It depends on the physicochemical properties of the DNA, mainly its charge and its flexibility characterized by the persistence length (50 nm for dsDNA and few nm for ssDNA in medium ionic strength). Consequently, the adsorption behavior of ssDNA and dsDNA molecules is radically different. Different spreading methods have been specifically adapted to either dsDNA or ssDNA observations. With regard to dsDNA, two main approaches have been distinguished, the “hypophase/hyperphase method” and the “adsorption method”. The first one is based on the formation of a cytochrome c protein film at the water–air interface in which DNA molecules are stretched. In fact, the film containing DNA molecules deposited on the surface of the water hypophase constitutes a hyperphase [5–9]. The second one, the “adsorption method” is based on the functionalization of the carbon surface by the deposition of sticky positively charged substances (Mg^2+^, Ethidium Bromide, PolyHistidine, PolyLysine, Spermidine or Pentylamine/Amylamine). The dsDNA, which is negatively charged, is then absorbed onto the surface [10–15].

The case of ssDNA spreading is more challenging as it naturally collapses onto the carbon surface due to its high flexibility and its folding into small secondary structures (hairpins). To analyze replication or recombination intermediates containing ssDNA regions, an alternative spreading method has been developed using a detergent film that stretches dsDNA, ssDNA, mixed ss–dsDNA molecules, and more complex DNA structures. The so-called BAC (benzyl dimethyl alkyl ammonium chloride) produces a floating film (hyperphase) that spreads out at the interface between water (hypophase) and air in which DNA molecules are embedded [16–19]. BAC remains the reference method commonly used to characterize replication intermediates. DNA samples are stained in presence of very low concentration of uranyl acetate (UrAc) followed by shadowing, which uses a vacuum-deposited heavy metal thin film (typically platinum/carbon) by rotary deposition. The sample is then observed in bright-field imaging mode.

For >30 years, our laboratory has developed TEM methods dedicated to the study of DNA conformation, both intrinsic and induced by proteins, with a specific expertise in the characterization of nucleoprotein machinery involved in DNA repair pathways, including HR [20–24]. For this, we have optimized the TEM methodology initially developed by Jacques Dubochet [17] consisting on the preactivation of a very thin carbon film with glow discharge in presence of pentylamine/amylamine (primary amine), followed by DNA spreading, and its staining with UrAc (from 0.02% to 2%). This approach gives rise to the positive staining of the sample, as DNA is decorated with fine clusters of UrAc, without staining of the carbon support. Interestingly, our previous atomic force microscopy thickness measurements (Z height) of dsDNA spread on carbon grid revealed a mean value of 3 nm. The height of the ssDNA could not be determined with precision, but is anyway <2 nm. In addition to staining the DNA, this method promotes the recruitment and the deployment of the DNA backbone onto the surface maintaining its 3D/2D properties [25, 26]. The primary amine stably anchored to the carbon film recruits the DNA molecules at different points, thus limiting their 2D diffusion. UrAc allows both their deployment and staining. As initially demonstrated by Dubochet et al. [10], it then allows DNA observation in dark-field mode, which is based on Z-contrast imaging that gives a direct image of atomic structural composition [27] (UrAc clusters in our method). We use annular or crystallographic dark-field mode and an energy filter to select zero-loss energy electron providing fine contrast [26, 28, 29]. Fine studies on DNA local flexibility, curvature, and topology have contributed to prove the relevance of this positive staining approach [26, 29–32], which is also suitable to the study of DNA–protein complexes involved in DNA metabolism pathways allowing to characterize the behavior of proteins on DNA, from their binding modes to recognition events [20–23].

In order to contribute to the studies of replication intermediates, we combined our direct adsorption approach with the BAC film method to optimize the recruitment of DNA onto the carbon surface, its spreading, and its positive staining under stretching conditions. We named this new approach BAC film positive staining (BPS). The present work describes the technical optimizations we have developed using a rapid and reproducible sample preparation procedure in order to characterize dsDNA as well as ssDNA, mixed ss–dsDNA and branched DNA from synthetic DNA substrates to *in vivo* DNA structures formed along DNA transactions. We also detail the methods to generate DNA substrates for reconstitution *in vitro* assays in the presence of purified proteins. We then provide procedures to characterize *in vivo* DNA intermediates from human cells, with a focus on the critical steps of the DNA isolation. Finally, in a practical case study, we present original observations of *in vivo* replication, recombination, and resection DNA intermediates highlighting the DDR pathways identified in cells undergoing two different genotoxic stresses.

## Material and methods

### Generation of DNA substrates

#### Synthesis of 5′ junction (with a 3′ ssDNA overhang) and gap substrate

Two DNA fragments of 1440 and 609 bp were amplified from pBR322 plasmid by PCR using *Taq* polymerase (NEB) and the pairs of primers Cy5-2574^+^ and biotin-4014^−^, biotin-2574^+^ and 3185^−^, respectively. The biotinylated PCR products were purified on a MiniQ 4.6/50 ion exchange column (GE Healthcare Life Sciences) and loaded onto a HiTrap Streptavidin HP column (GE Healthcare Life Sciences). Purification of the nonbiotinylated strand was achieved by elution with 80 mM NaOH, neutralized by addition of HCl 1 M and annealed at equimolar concentrations in molecules, in presence of 1.5 mM MgCl_2_ then purified on anion exchange MiniQ column.

For the ds–ss–ds, in this article, mentioned as gap substrate, three DNA fragments of 400, 600, and 1440 bp were amplified from pBR322 plasmid by PCR using the pairs of primers biotin-2574^+^ and Cy5-2974^−^ (400 bp), biotin-3413^+^ and Cy5-4014^−^ (600 bp), and 2574^+^ and biotin-4014^−^ (1440 bp). The three nonbiotinylated ssDNA fragments were purified by elution on HiTrap Streptavidin HP column and annealed at equimolar concentrations in molecules as described then purified on the anion exchange MiniQ column mentioned above.

#### In vitro *Holliday junction synthesis*

A 660 nt-long sequence was amplified from PBR322 plasmid using one biotinylated primer and one a nontagged primer to generate two different amplicons named as X1 and X2 (Supplementary Fig. 1). These primers were designed to add two different additional sequences of 19 nt located in 3′ (named as mod1 and mod2) necessary for the further final structure assemblage. In parallel, two identical biotinylated fragments were generated using primers designed to localize the mod1 and mod2 sequences in the 5′ of the amplicons, named X3 and X4, respectively. Then the four amplicons were loaded on a HiTrap Streptavidin HP column in order to purify the nonbiotinylated strands following manufacturer’s instructions. After a transient denaturation (95°C for 5 min), the X1 fragment was slowly annealed with its antiparallel counterpart X2 (and X3 with X4) at equimolar concentrations, overnight at RT in presence of 1.5 mM MgCl_2_. This generated two pseudoforks with mod1 and mod2 ssDNA overhangs located either in 5′ or 3′. Both were assembled following heating at 50°C for 30 min then annealing at RT for 2 h to generate selective hybridization between both mod1 and mod2 extremities. Local strand spontaneous slippages generated the suited HJ structures with various arms lengths.

## Homologous recombination *in vitro* assays

### Protein purification for *in vitro* assays

*Saccharomyces cerevisiae* replication protein A (RPA) heterotrimer and Rad51 proteins were purified as previously described [33–36]. Yeast Rad54 was purified as previously described [24].

### Displacement loop assay

The reaction contained 10 mM Tris-HCl pH7.5, 50 mM NaCl, 3 mM MgCl_2_, 1.5 mM ATP, 1 mM DTT, 10 mM phosphocreatine, 35 U/ml phosphocreatine kinase, 25 nM molecules (36 µM nt) of 5′ junction DNA with overhang labeled with Cy5. Rad51 at a final concentration of 12 µM (1 protein per 3 nt) was introduced into the reaction and incubated 10 min followed by RPA addition at a final concentration of 1.44 µM (1 protein per 25 nt) also during 10 min. Rad54 and homologous pUC19 dsDNA plasmid (purchased from NEB and purified on MiniQ ion-exchange chromatography column) were added together in a final concentration of 175 nM and 25 nM in molecules of dsDNA (seven proteins per dsDNA molecule) in a final volume of 14 µl during 20 min. This assay was carried out at 30°C. In these reactions lacking Rad54 (control), storage buffer was added in order to rule out nonspecific effects of buffer components and to maintain the ionic strength in all samples. For agarose gel analysis, 7 µl of the 14 µl reaction was taken and stopped using 0.5 mg ml^−1^ Proteinase K, 1% sodium dodecyl sulfate (SDS), 12.5 mM EDTA and incubated overnight at room temperature. A 1% TAE agarose gel was run at 70 V, for 40 min.

### Strand exchange assay

13 μM Rad51 were incubated with 40 µM of circular viral ssDNA (+) PhiX174 (previously purified in ion-exchange MiniQ column) for 5 min, in 10 mM tris-HCl pH 7.5, 100 mM NaCl, 3 mM MgCl_2_, 2 mM ATP, 1 mM DTT, and 2 mM Spermidine, and 1.3 µM RPA was then added into reaction for 15 min. Twenty micrometers of linear dsDNA PhiX174 replicative form I were added into the reaction for 50 min (unless stated), to a 10 µl final volume. The dsDNA was linearized by digestion with the restriction enzyme XhoI or PstI (NEB) followed by phenol–chloroform extraction. The reaction was performed at 37°C. Reactions were cross-linked with trioxsalen (final concentration 10 µg/ml) (Sigma) during 4 min under UVA (cross-link density of 1 per 200–300 nt) and stopped using 0.5 mg/ml proteinase K, 1% SDS, 12.5 mM EDTA, and incubated overnight at room temperature. Samples were separated on a 0.8% D5 agarose (Condalab, Dutscher) gel electrophoresis in TAE 1×, run at 40 V for 2.5 h and finally stained with SYBR gold (Invitrogen). For TEM analysis, four independent *in vitro* strand exchange reactions were pooled, and then DNA was purified using phenol–chloroform and ethanol precipitation.

## Isolation of replication intermediates from human cells

Cells were seeded at 3.10^6^ in 100 mm dishes and potentially subjected to UVC irradiation (15 or 25 J/m^2^ followed by 1 h at 37°C) or camptothecin (CPT) (200 nM, 1 h at 37°C) treatments the day after. Then asynchronous and nonconfluent cells were incubated with 10 ml PBS supplemented with 10 µg/ml trioxsalen (Sigma) for 5 min on ice covered from light. Following a 45 kJ UVA irradiation, cells were then subjected to another round of trioxsalen/UVA irradiation. After a PBS wash, cells were scrapped, washed, and centrifuged, then nuclei were isolated in order to avoid cytosolic material contaminations following manufacturer’s instruction (Nuclei EZ prep, Sigma). The genomic DNA (gDNA) was extracted from nuclei using DNAzol (1 ml/dish) (Invitrogen) that removed most of the chromatin-bound proteins and residual RNA. Then gDNA was precipitated by adding 0.5 ml 100% ethanol, washed in 70% ethanol and finally resuspended in water, avoiding the use of vortex, micropipette, or centrifugation. The material was subjected to proteinase K (1 mg/ml) hydrolysis overnight at 55°C in the presence of 10 mM Tris-HCl, 20 mM NaCl, 1 mM EDTA, and 1% SDS. After a smooth phenol/chloroform extraction, the gDNA was subjected to a second round of proteinase K hydrolysis and phenol/chloroform extraction. Following the precipitation with 0.3 M sodium acetate and 2.5 volumes of 100% ethanol, the gDNA was washed in 70% ethanol and resuspended in water. The gDNA was quantified by NanoDrop (Thermo). Then it was fragmented by enzymatic restriction using 10 U/µg DNA BamHI or 5 U/µg EcoRI for 2 h or overnight in presence of excess amounts of RNase A to hydrolyze all forms of remaining nuclear RNA in low-salt conditions. After supplementation with 1 mM EDTA and 300 mM NaCl, the gDNA was loaded onto poly-prep chromatography column (Bio-Rad) stacked with benzoylated naphthoylated DEAE–Cellulose (Sigma) as previously described [37]. The first four ssDNA-containing fractions were pooled, precipitated, quality controlled by agarose gel analysis, and finally spread for TEM analysis.

## Evaluation of ssDNA accumulation through the monitoring of RPA70 recruitment

Human fibroblasts (MRC5-V1) cells were cultured at 37°C in MEM (Gibco) supplemented with 10% fetal calf serum, 100 U/ml penicillin and 100 µg/ml streptomycin under 5% CO_2_. Cells were seeded at 4.10^5^ cells per well in six-well plates, subjected to indicated treatments the day after, and harvested by trypsinization at indicated time points. Cells were washed in PBS and centrifuged, then pellets were resuspended in 1 ml/well of CytoSKeleton 100 buffer (10 mM PIPES pH 6.8, 100 mM NaCl, 300 mM sucrose, 3 mM MgCl_2_, 1 mM ethylene glycol tetraacetic acid, 0.2% Triton ×100, anti-proteases) and incubated 15 min on ice. Samples were then centrifuged at 7000 rpm for 5 min at 4°C. Pellets were then resuspended in 100 µl of benzonase buffer (10 mM Tris pH 7.5, 20 mM NaCl, 1 mM MgCl_2_, 0.4% SDS, anti-proteases, 0.15 U/µl benzonase) and incubated 30 min at 37°C. Protein quantity was evaluated using NanoDrop. After denaturation in Laemmli buffer at 90°C for 10 min, the proteins were run on 10% sodium dodecyl sulfate–polyacrylamide gel electrophoresis (SDS-PAGE). Membranes were finally blotted with antibodies directed against RPA70 (Calbiochem) or H3 (Abcam) proteins.

## TEM for DNA intermediates imaging

### Preparation of carbon-coated grids

TEM copper grids 600 mesh (EMS) were covered with a very thin carbon film (2–4 nm), which functions as a support surface for sample deposition and spreading. The contrast increases significantly with the fineness of the carbon film. To prepare carbon films of a few nanometers thick, a carbon thread (Bal-Tec) was evaporated onto a freshly cleaved mica sheet (EMS) in the vacuum evaporator (MED10/MED20 Balzers/Bal-Tec) at distance of about 100 mm from the carbon source at 10^−4 ^Pa. Then the carbon-coated mica was slowly plunged in a MilliQ water pool to separate the carbon from the mica, and the floating carbon film was deposited onto the grids, previously set at the bottom of the dish by a slow water flow out. Finally, the carbon-coated grids were dried.

### Functionalization of the carbon-coated grids

**Figure 1:**
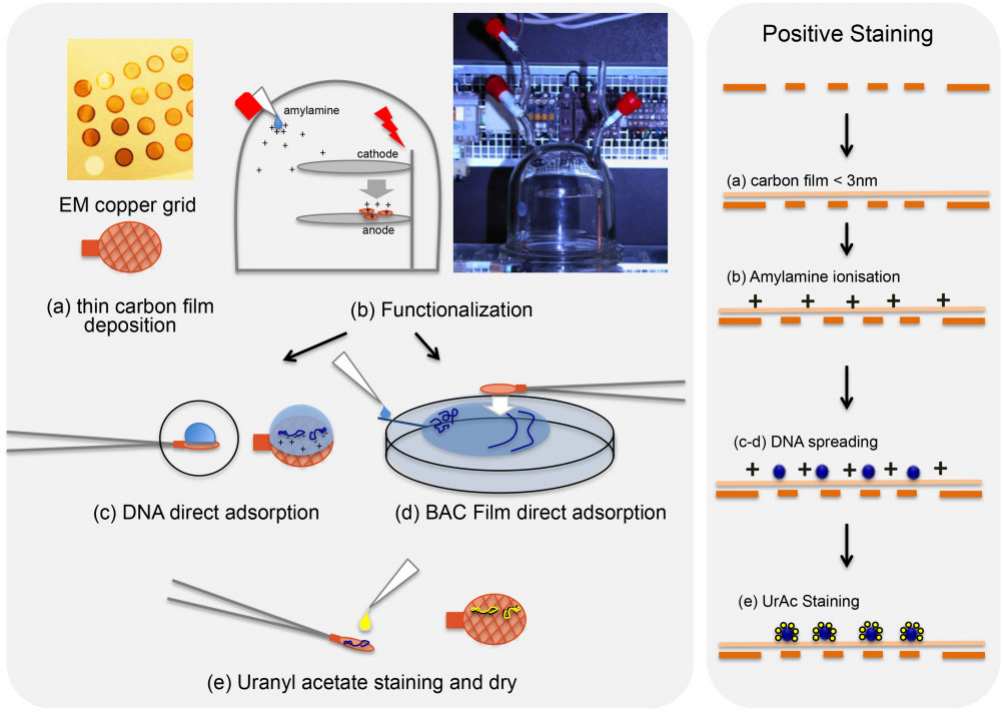
Two experimental procedures for nucleic acids sample preparation and imaging. (**a**) Deposition of a thin carbon film on the TEM copper grid. Direct adsorption method by (**b**) functionalization by evaporation of the positively charged amylamine agent under high voltage in the device held under vacuum, then (**c**) DNA direct adsorption after 5 µl sample drop (concentration 0.5 µg/ml) deposition, and finally (**e**) positive staining using UrAc, rinsing, and dry. BPS method by (**d**) deposition and spreading of 2.5 µl of DNA sample (concentration 25 µg/ml) in a BAC hypophase/hyperphase film formed at the interface between water and air, then adsorption on an amylamine pretreated grid, and finally (e) positive staining using UrAc and dry.

The functionalization of a very thin (2–4 nm) carbon film coating grids was carried out by pentylamine or amylamine (Sigma Aldrich) ionization using a glow discharge, providing stable NH+ charges deposition onto the carbon surface (Fig. 1) [28, 29]. Grids were deposited on a glass slide coated with parafilm and placed on the lower steel plate of our lab-made device (Fig. 1). It consists of a glass bell in which the vacuum is generated by a vacuum pump and an inlet allows the controlled introduction of pentylamine/amylamine, which is vaporized under high voltage. At 2.6 Pa vacuum (0.026 mbar), a drop of amylamine is introduced up to about 26.7 Pa (0.27 mbar), and then ionized over 30 s by switching on the tension (900 V) to form a plasma. Grids prepared in this manner keep functionalized properties for about 1 day.

### Direct adsorption spreading method for canonical positive staining

Five microliters of DNA or DNA–protein complexes as indicated (at 0.5 µg/ml DNA) were deposited on the carbon-coated grid for 1 min and then rinsed with two drops of a 2% (w/vol) solution of aqueous UrAc which allowed a fine staining of DNA molecules [27, 28]. After a careful drying with an ashless filter paper (Macherey-Nagel, VWR), the grids were directly subjected to TEM analysis.

#### BAC film hyperphase associated with positive staining

Fresh cleaved mica sheet (rectangles of 1 cm per 2 cm) was partially immersed (45° angle) in a petri dish with 14 cm diameter containing MilliQ water. Two separated drops were deposited in a 1.5 ml Eppendorf tube: a drop containing 2 µl of formamide (Sigma Aldrich), 0.8 µl of 10% BAC (benzyl dimethyl alkyl ammonium chloride) (Sigma Aldrich) and 0.4 µl of glyoxal (Sigma Aldrich), and another drop containing 2.5 µl of ultrapure DNA sample at 25 µg/ml. Just before initiating the spreading, some graphite powder was loaded on the water surface near the mica sheet in order to visually delimit the hyperphase film. After the two drops were quickly spun down forming the hyperphase, the mix was loaded on the mica, slipped along the ramp and therefore onto the water surface, creating the spreading film. The DNA was picked up on functionalized carbon-coated grids by briefly touching the film surface. Grids were washed in a large volume of purified MilliQ water and then 100% ethanol and dried with filter paper. Finally, grids were stained with three drops of 2% UrAc and dried.

### Tem imaging

Samples were visualized in annular or crystallographic dark-field mode using Zeiss TEM902 or Zeiss TEM912 microscopes, respectively. TEM902 was equipped with a magnetic prism electron filter and TEM912 with an Omega energy filter allowing energy-filtered imaging. Such imaging techniques use the energy loss spectrum properties to increase the contrast. In dark-field mode, only electrons scattered at wide angles are collected while the central beam is eliminated. This mode was obtained using either an annular condenser aperture combined with an objective aperture of 40 µm (for Zeiss 902) or a tilted beam combined with an objective aperture of 60 µm (for Zeiss 912). Finally, only zero-loss electrons were selected (zero-loss filtering), which prevents inelastically scattered electrons from contributing to image formation and improves image contrast and resolution. Electron micrographs were acquired using a Veleta or Tengra high-resolution 2k × 2k CCD camera associated with a dedicated iTEM software (Olympus, Soft Imaging Solutions) at ×30 000 to ×140 000 magnification.

## Results and discussion

### DNA visualization using direct adsorption compared to BPS

Our direct absorption method associated with positive staining allows DNA molecules to deploy rather than being projected on the surface of the carbon film, thus maintaining their conformational properties. In these conditions, the DNA adsorption properties are independent of the presence of counterions, both monovalent (Na^+^/K^+^) or divalent (Mg^2+^ or Mn^2+^) in buffer solutions. Indeed, DNA conformation and/or topology can be analyzed in various conditions of ionic strength, pH, and in the presence of ligands. Digitizing and contour tracking allows calculating local curvature and flexibility along large linear DNA molecules or on small ones containing specific sites such as a curved region, single-strand break, or abasic site using the appropriate algorithms [20, 26, 30, 31, 38, 39]. This approach can be extended to closed circular plasmids, minicircles, or for the characterization of nucleoprotein machineries related to DNA repair pathways, including HR [21–24, 29, 32, 39–43]. The quality of the positive staining is directly related to the quality of the amylamine-mediated functionalization of the thin carbon film using our lab-made device (see Material and methods), giving a slight decoration of the DNA by UrAc with almost no UrAc background contamination. Due to the fineness of the staining, the sample must be visualized in dark-field imaging mode, which allows a very fine contrast, each cluster of UrAc deposited along the DNA being directly detected [27]. This procedure avoids an additional step of sample shadowing which usually increases the contrast but leads to the artificial thickening of DNA and loss of resolution [28].

**Figure 2:**
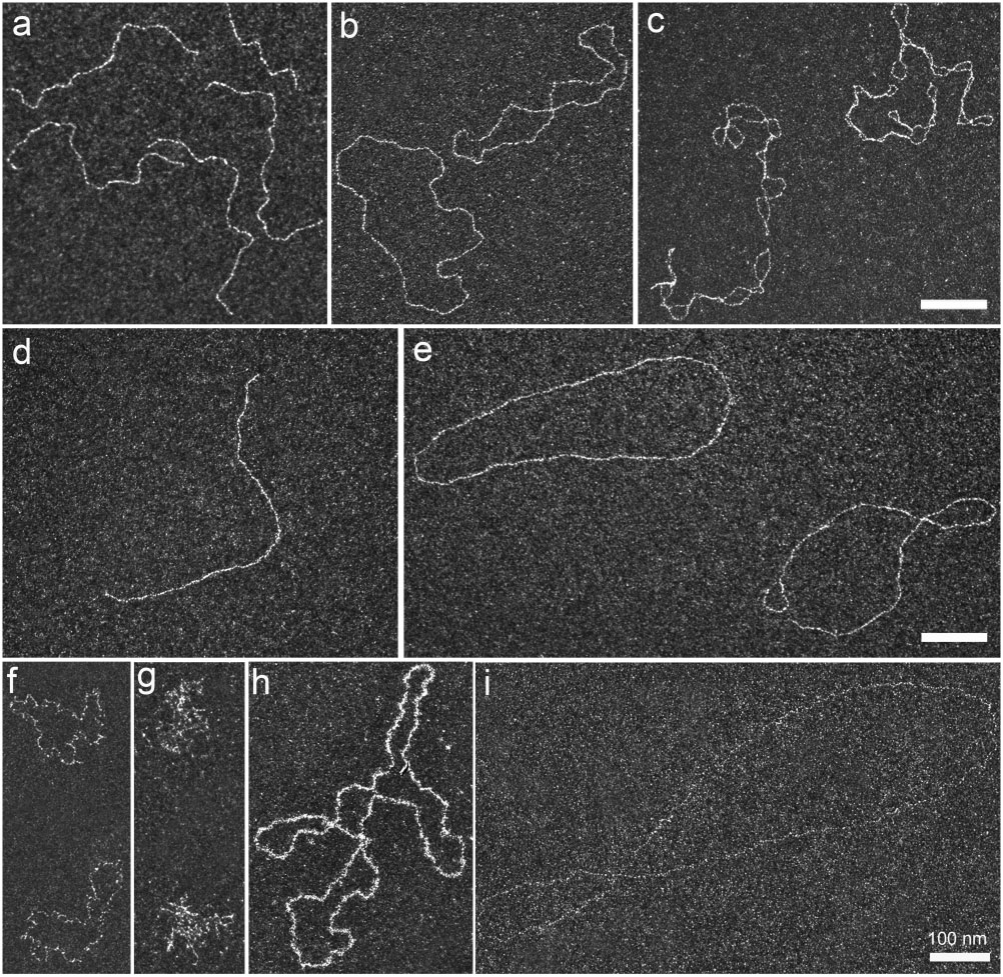
Ds and ssDNA visualization in dark-field imaging mode using both direct adsorption and BPS methods. (**a**) Positive staining of a linear dsDNA fragment of 1440 base pairs (bp) visualized in dark-field mode. (**b**) Toroidal form of closed negatively supercoiled pUC19 dsDNA plasmid obtained after dilution in a 10 mM Tris-HCl pH 7.5 buffer without salt. (**c**) Usual plectonemic form of closed negatively supercoiled pUC19 dsDNA plasmid diluted in 10 mM Tris-HCl, pH 7.5 buffer in presence of 50 mM NaCl. (**d**) Linear 1440bp dsDNA fragment and (**e**) negatively supercoiled pUC19 plasmid spread using the BPS combined methods. Linear and closed circular DNAs are stretched onto the surface. (**f**–**i**) Positive staining of ssDNA PhiX174 virion spread in water (f) and in a buffer containing 10 mM Tris-HCl pH 7.5, 50 mM NaCl (g). In the latter buffer, ssDNA is folded due to its high flexibility and secondary structures formation. (h) SsDNA PhiX174 virion can be revealed after incubation with *E. coli* SSB proteins (one protein per 20 nucleotides in buffer 10 mM Tris-HCl pH 7.5, 50 mM NaCl). The reaction is diluted before its spreading using direct adsorption method. (i) SsDNA PhiX174 virion, spread using BPS method ssDNA appears very thin and stretched onto the surface. All scale bars represent 100 nm.

Linear 1440 bp dsDNA fragment and closed negatively supercoiled pUC19 plasmid were first visualized in dark-field imaging mode after their direct absorption and positive staining **(**Fig. 2a–c**)**. Interestingly, we were able to demonstrate the reversible transition from the toroidal to plectonemic form of negatively supercoiled DNA without any change in the topological constraints (no ssDNA break) **(**Fig. 2b and c, respectively**)**. Toroidal form of supercoiled DNA was obtained at very low ionic strength (i.e., in absence of counterions), the mutual repulsion of the negative charges of the phosphate backbone conferring rigidity to DNA. Addition of monovalent salt restored the canonical plectonemic form of the plasmid without altering its adsorption properties. PhiX174 ssDNA virion diluted in water and spread by direct adsorption and positive staining, appeared to be partially deployed (Fig. 2f**)**, while the addition of counterions caused its collapse on the surface due to its high flexibility and its folding with the formation of secondary structures (Fig. 2g**)**. Covering ssDNA with specific ssDNA-binding (SSB) proteins was another way to identify and characterize ssDNA (Fig. 2h).

To develop the analysis of ssDNA containing intermediates, we combined this direct absorption with the usual BAC film hyperphase **(**Fig. 1**)**. It allowed us to take advantage of the BAC film for stretching the intermediates and of the positive staining for their recruitment, their staining and their observation in dark-field imaging mode. Beforehand, we concluded that amylamine is the best positively charged agent to provide DNA recruitment, compared to Mg^2+^, Ethidium Bromide, PolyHistidine, PolyLysine, or Spermidine pretreatment, giving the best quality of positive staining (data not shown). This combination allowed the deployment and stretching of dsDNA (Fig. 2d and e), linear 1440 bp dsDNA fragment and negatively supercoiled pUC19 plasmid being stretched onto the surface, until the plectonemes of pUC19 totally disappeared through a redistribution of Twist and Writhe. The ssDNA PhiX174 was also clearly stretched using this BPS approach (Fig. 2i**)**. Importantly, we observed that the BAC film integrity could be affected by the presence of proteins or other contaminants remaining in the DNA sample. With regard to the imaging conditions, we noticed that the positive staining is slightly affected using BPS compared to direct adsorption. First, the background appeared slightly brighter probably due to the interaction between small UrAc clusters with the BAC film on the carbon surface. Second, DNA stretching reduces UrAc staining, which makes DNA thinner. The dark-field mode is essential for such imaging.

## TEM analysis of synthetic DNA intermediates

Synthetic ss–dsDNA substrates (1–3 kb) designed for replication/recombination *in vitro* investigations can be efficiently analyzed using both direct absorption and BPS approaches.

### Mixed ss–ds and branched DNA substrates

**Figure 3:**
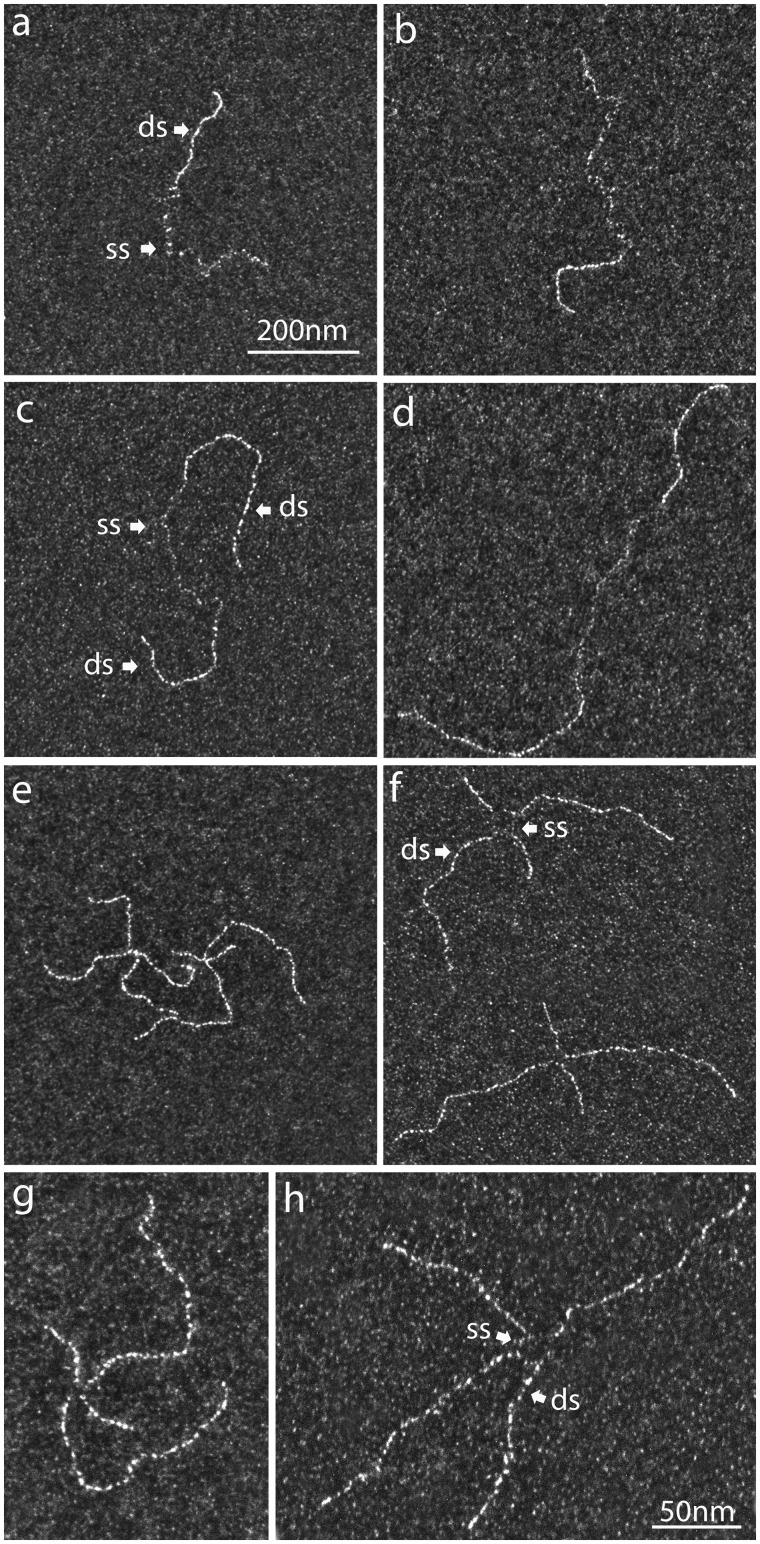
DNA substrates characterization using direct adsorption and BPS method. (**a**, **b**) Comparison of a 5′ junction ss–dsDNA substrate spread using direct adsorption and BPS methods, respectively. The dsDNA is spread in the same way while the ss part is stretched using the BPS method. (**c**, **d**) Comparison of an ssDNA gap contained in dsDNA substrate spread using direct adsorption and BPS methods, respectively. (**e**, **g**, **f**, **h**) Comparison of a HJ DNA substrate spread using direct adsorption and BPS methods, respectively. This synthetic HJ displays a bubble at the crossing point. The magnification is the same (×85 000) for each picture, (a–f): the scale bar represents 200 nm; (g–h) the scale bar represents 50 nm.

We set up protocols to design and prepare ss–ds hybrid and ds–ss–ds (gapped) DNA molecules that could be used to mimic part of DNA intermediates, such as postreplicative gaps formed during replication, and other recombination intermediates. We prepared dsDNA linear fragments extended with a long single-stranded overhang (3′ or 5′) mimicking the double-strand break after the resection step on the early stages of HR (see “Following HR intermediates using a TEM-suitable D-loop assay” section) (Fig. 3a and b), or single-stranded gaps that mimic repriming events during replication (Fig. 3c and d). The substrates were spread using both direct adsorption and BPS methods and visualized in dark-field mode (Fig. 3 panels of left and right, respectively). The ss and ds regions of the substrates were easily distinguishable under both conditions although the analysis of ssDNA within ss–ds hybrid and ds–ss–ds (gap) was clearly facilitated with the BPS method thanks to its stretching. These substrates were helpful to highlight the specific binding of a protein to the ss–dsDNA junction and can be used in *in vitro* HR experiments [23, 24]. Moreover, they may also be used to test working hypothesis concerning gap-filling mechanisms by comparing the structures obtained *in vitro* with those from *in vivo* experiments.

In a similar approach, we constructed synthetic four ways DNA substrates also known as Holliday Junctions (HJ) (Fig. 3e–h). BPS spreading revealed that all four-ways molecules clearly exhibited a denaturation bubble located at the junction point (“open centered structures”), rarely observed using direct adsorption spreading.

TEM quantitative analysis demonstrated that these DNA substrates were never destructured upon both spreading methods.

### Following HR intermediates using a TEM-suitable displacement loop assay

HR uses a DNA template to repair damaged DNA by finding and copying the intact homologous DNA sequence of the sister chromatid. Rad51 recombinase forms an ATP-dependent presynaptic helical filament on ssDNA resulting from resection and able to catalyze homology search, strand invasion, and strand transfer [44–47]. We have developed *in vitro* approaches to generate and identify specific HR intermediates formed during homology search, strand invasion, and strand transfer mechanistic steps.

**Figure 4:**
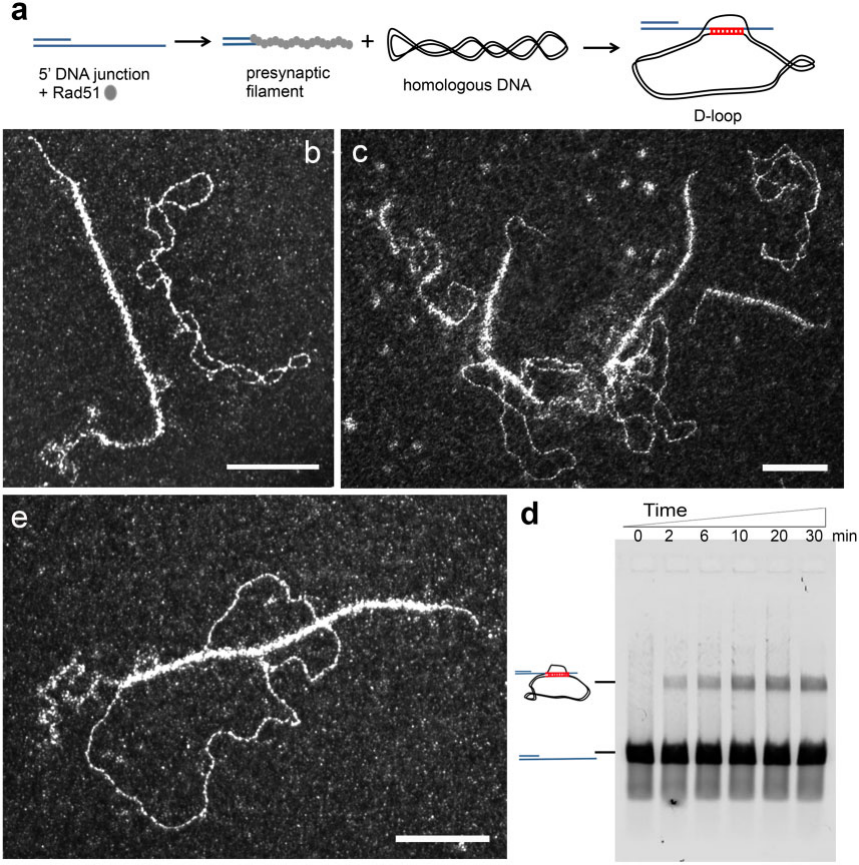
D-loop assay followed by TEM in dark-field mode using direct adsorption method. (**a**) Scheme of the D-loop reaction. The 5′-DNA junction was incubated with Rad51 (one protein per three nucleotides) for 3 min followed by RPA addition (1 protein per 60 nt) 10 min at 37°C in a buffer containing 10 mM Tris-HCl (pH 8), 50 mM NaCl, 3 mM MgCl2, 1 mM DTT, and 1.5 mM ATP. Then homologous dsDNA pUC19 negatively supercoiled plasmid was added to the reaction during 30 min. RAD51 polymerizes onto the DNA substrate to form the presynaptic filament and then catalyzes the formation of the synaptic complex with the homologous DNA. (**b**) TEM representative view of the presynaptic filament formed on the 5′ junction and the homolog plasmid. (**c**) Representative view of the reaction (20 min). (**d**) Zoom on a synaptic complex (joint molecule formed between the presynaptic filament and the homolog plasmid); (**e**) half of the reaction was deproteinized then run on an electrophoretic gel, the D-loop band is visible within 2 min following homolog plasmid addition. All scale bars represent 100 nm.

The nucleoprotein filament performs homology search by probing duplex DNA throughout the whole genome. Mechanistically, when homology is found, the interaction between the nucleoprotein filament on ssDNA and the duplex DNA donor results in their incorporation into a three-stranded intermediate, the synaptic complex [48–50]. Then the invading ssDNA and its complementary DNA strand in the dsDNA donor intertwine, forming the heteroduplex DNA and after the third strand is displaced, yielding a structure called the displacement loop (D-loop) [51, 52]. The D-loop is an important HR intermediate since its formation is required to prime DNA synthesis [33] but its precise architecture is still poorly known. To reconstitute HR synaptic intermediates such as D-loop and gain molecular insight, we have set up a D-loop assay and followed the intermediates formed along the reaction using direct adsorption of the sample and TEM analysis (Fig. 4). Rad51 (from yeast or human) was incubated with a 5′ junction DNA substrate to form the presynaptic filament, and then a homologous supercoiled dsDNA plasmid was added (Fig. 4a and b). Rad51-mediated synaptic complexes are the protein-containing joint molecules resulting from the interaction of Rad51–ssDNA with the homologous dsDNA donor. We were able not only to quantify the amount of joint molecules, but also to evaluate the length of the contact zone, to assess the presence of proteins on it (with the ability to specifically label proteins using gold bead coupled antibodies), and finally to characterize the topological properties of the homologous dsDNA donor (Fig. 4c and d) [24]. The traditional D-loop assay involves deproteinization of the reaction products, allowing the DNA species separation by electrophoresis and the reaction yield quantification along time **(**Fig. 4e**)**.

### Structural characterization of DNA intermediates formed during DNA strand exchange

**Figure 5:**
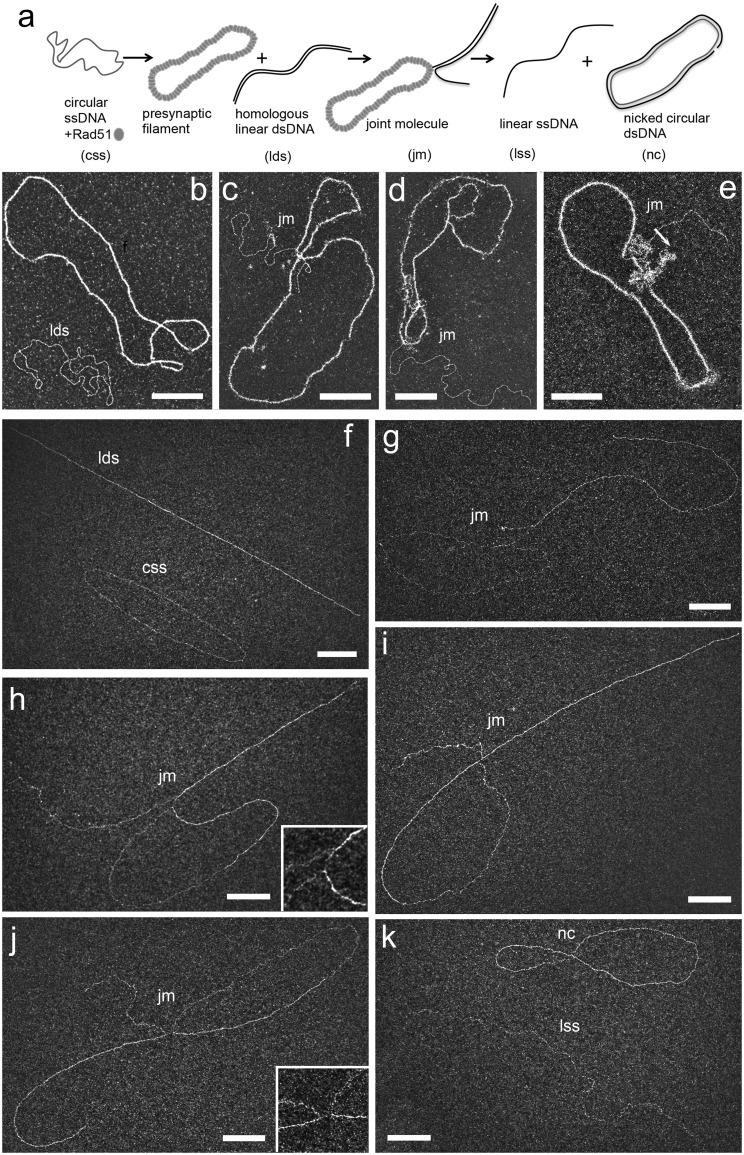
Strand exchange assay followed by TEM using direct adsorption or cross-linking/deproteinization combined with BPS spreading. (**a**) Scheme of the strand exchange reaction. The ssDNA PhiX174 virion (named circular ssDNA, css) was incubated with Rad51 (one protein per three nucleotides) for 3 min followed by RPA addition (1 protein per 60 nt) 10 min at 37°C in a buffer containing 10 mM Tris-HCl (pH 8), 50 mM sodium chloride, 3 mM Magnesium chloride, 1 mM DTT, and 1.5 mM ATP. Then linearized PhiX174 RFI homologous plasmid (named linear dsDNA, lds) was added to the reaction during 30 min. RAD51 polymerizes onto the ssDNA substrate to form the presynaptic filament and then catalyzes the strand invasion and strand transfer with the linear homologous dsDNA. Final products are linear ssDNA (lss) and nicked dsDNA plasmid (nc). (**b**–**e**) The reaction is diluted and spread using direct adsorption method followed by positive staining. (b) DNA substrates at the beginning of the reaction (presynaptic filament formed by Rad51 polymerization on css and naked lds); (c–e) representative joint molecules; (**f**–**k**) The reaction is cross-linked using psoralen/UVA irradiation, deproteinized, DNA intermediates are extracted using phenol/chloroform then diluted and spread following BPS procedure. (f) DNA substrates at the beginning of the reaction after deproteinization (css and lds); (g–j) representative joint molecules formed during the reaction (20 min). The first contact between the circular ss- and ds-DNA occurred through the extremity of the linear dsDNA (g). A small part of the lds has been transferred to the css. An ssDNA region (“gap”) is often visible at the point of strand transfer (see zooms on (h) and (j)). (**k**) Final products of the reaction: linear ssDNA (lss) and nicked circular dsDNA (nc). All scale bars represent 200 nm.

The DNA strand exchange assay is mechanistically different from the D-loop assay, mainly because of the long and fully homologous DNA substrates used which allow the complete strand transfer. Recombinases promote *in vitro* strand exchange between circular ssDNA and linear homologous dsDNA by catalyzing the strand transfer from the extremity of the linear dsDNA to the circular DNA strand originally unpaired [53, 54]. The presynaptic filament was performed by Rad51 polymerization on PhiX174 circular ssDNA in the presence of RPA, then the previously linearized homologous dsDNA PhiX174 was added (Fig. 5a). The formation of joint molecules by Rad51 between circular ssDNA and linear dsDNA and further final products (nicked circles) were monitored by TEM analysis providing mechanistic details (Fig. 5b–k**)**. After addition of the donor dsDNA to the preformed presynaptic filaments, direct adsorption spreading of the sample revealed the formation of different joint molecules due to the capture of linear dsDNA by the Rad51 filament on circular ssDNA (Fig. 5c–e), leading to different lengths of strand transfer within the filament, with formation of displaced ssDNA covered by RPA (cloudy region, near the arrow pointing the strand transfer region). As shown in Fig. 5d, the first contact between the circular ss- and ds-DNA only occurred through the extremity of the linear dsDNA. To better reveal the architecture and to allow a more precise analysis of the intermediates, joint molecules must be deproteinized and deployed on the surface. In that way, samples were stabilized using UVA/trioxsalen *in vitro* cross-linking, deproteinized and subjected to BPS and TEM analysis (Fig. 5f–k). We identified two populations of joint molecules: one where the DNA strand transfer was initiating through the extremity of the linear dsDNA containing a small portion that had been transferred (Fig. 5g**)**, and a second exhibiting a longer length of transferred strand **(**Fig. 5h–j**)**. Notably, in most of them, ∼50% of the total length of linear dsDNA had been transferred. These two intermediates seemed to be kinetically stable joint molecules during the strand exchange reaction. Interestingly, in some joint molecule intermediates, we detected the presence of ssDNA region at the transfer zone (Fig. 5h and j). We inferred this happens because, as proposed for RecA, a segment of the exchanged strand is maintained in a three-stranded complex with Rad51, which is disrupted upon deproteinization [48, 55, 56].

## Characterization of *in vivo* DNA intermediates

Various endogenous and exogenous damages induce replication fork stalling and collapse, a source of genomic instability. The study of DNA intermediates generated at replication fork represents a crucial point to understand how the replication machinery coordinates with DNA repair and Damage Tolerance (DDT) mechanisms. Efforts have been made to decipher replication dynamics at the molecular level (e.g., DNA fiber assays), but structural evidences are often lacking. TEM constitutes a unique tool allowing the fine characterization of *in vivo* intermediate architectures. José Sogo, Jack Griffith, and David Dressler have been among the leaders in this field, developing TEM tools that gave access to the nucleic structures, thereby contributing to major advances on DNA replication and DDR understanding: the identification of HJs in *Escherichia. coli*, fork reversal in yeast and humans, replication fork stalling and fork restart downstream the DNA lesion (“repriming”), template switching in yeast, meiotic HR, but also identification of the till then theoretical telomeric loop (T-loop) in humans [57–64]. TEM procedures have been more recently updated notably by developing methods dedicated to human cell studies [37, 65, 66]. By combining an optimized *in vivo* sample preparation of these fragile structures to the BPS procedure, we improved their fine visualization.

### DNA intermediates preparation

In order to stabilize the structures, cells were first subjected to UVA/trioxsalen *in vivo* cross-linking, which generates interstrand DNA covalent link (interstrand cross-link) into gDNA sites that are not protected by the presence of nucleosomes or other chromatin-associated proteins. This procedure avoids further slippage or denaturation of DNA intermediates upon the subsequent sample preparation [67–69]. The efficiency of the cross-link was calibrated by transient denaturation as previously described [70] (data not shown). The second step corresponds to the subsequent purification of gDNA that requires a careful handling to avoid DNA breakages. Since genotoxic treatments specifically increase ssDNA accumulation, the gDNA material becomes more fragile and breakable. Concretely, this can generate asymmetric replication forks [61]. In a third step, a rigorous protein removal was carried out to remove tightly bound chromatin-associated proteins, in order to avoid the formation of nonspecific structures (see below) but also to maintain the hyperphase film integrity. In a fourth step, the gDNA was fragmented using enzymatic restriction, together with residual RNA removal to avoid its misleading with ssDNA and subsequent misunderstanding of the structures. Nuclei isolation and the use of DNAzol first optimized the RNA removal. Note that the efficiency of enzymatic fragmentation had to be set up depending on the structures of interest (choice of enzyme, enzyme/DNA ratio, and time of reaction). For example, excessive fragmentation generated short molecules but increased the enrichment specificity, i.e., reducing amount of molecules devoid of ssDNA. Conversely, mild fragmentation increased the rate of large molecules containing a unique/short ssDNA region. In a final step, the sample was then loaded onto a BND-cellulose chromatography column (a resin highly affine for ssDNA) allowing the separation of ssDNA containing molecules from the strictly dsDNA material by elution with caffeine agent. As structures containing ssDNA regions, DNA intermediates were enriched in caffeine fractions [37, 61, 71]. As a quality control, the enrichment efficiency was evaluated by TEM analysis of all fractions.

### Accurate characterization of *in vivo* DNA intermediates

**Figure 6:**
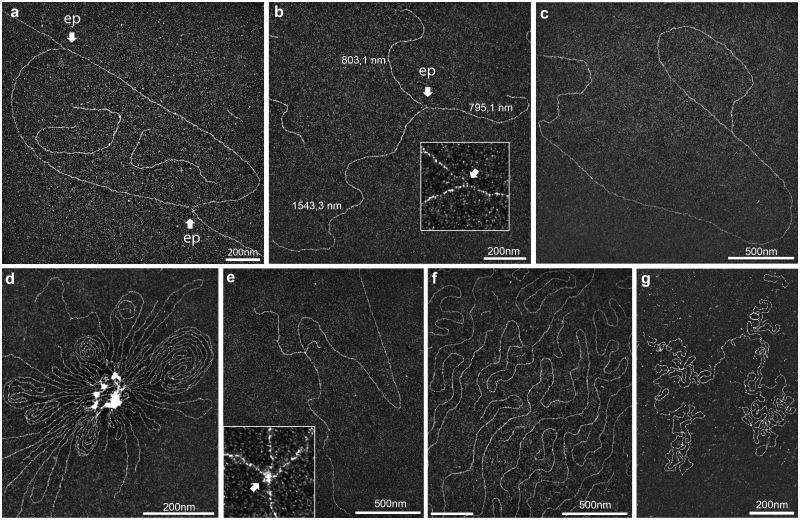
Reliable characterization of *in vivo* DNA intermediates. Replication intermediates were isolated from human fibroblastic cells as described and subjected to BPS method. (**a**) Typical replication bubble (untreated cells). (**b**) Typical replication fork resulting from the symmetric enzymatic digestion of a replication bubble, displaying two daughter strands of the same length and no visible ssDNA regions (untreated cells). (**c**) Loop-ended linear dsDNA molecule potentially corresponding to telomeric structures, or “T-Loop”, as described by Griffith *et al*. in 1999 (untreated cells). (**d**) An improperly deproteinized sample exhibited gDNA aggregation and protein clusters. (**e**) Subtle amounts of residual proteins (inlay) bridged two individual molecules together and consequently generated intermolecular crossings, generating nonspecific four-ways structures (untreated cells). Comparison of gDNA spread using BPS (**f**) and direct adsorption (**g**) methods. The BPS avoids random crossovers of long DNA molecules.

DNA intermediates were extracted from human fibroblastic cells, then spread using BPS approach and analyzed in dark-field imaging mode (Fig. 6). As transitory and dynamic entities, entire replication bubbles were rarely observed, because they quickly underwent fragmentation owing to the sample preparation (Fig. 6a). This hydrolysis resulted in symmetric replication forks after enzymatic cleavage of the two daughter strands (Fig. 6b). In untreated conditions, no accumulation of ssDNA in the vicinity of the fork was observed. Interestingly, loop-ended dsDNA molecules were sometimes identified (Fig. 6c) probably corresponding to telomeric structures (T-loops) previously evidenced using TEM [64].

As the structures of interest were expected to display branched DNA structures, accidental inter- and intramolecular crossovers have to be identified and limited. Performing BAC spreading avoided most of the dsDNA crossovers (compare Fig. 6f to Fig. 6g). However, the presence of residual chromatin-bound proteins can bridge multiple DNA molecules together **(**Fig. 6d**)**. Using our imaging mode allowed a fine identification of protein clusters appearing as a local increased contrast at the crossing point of two independent molecules (Fig. 6e).

### Characterizing DNA intermediates to identify the DNA damage tolerance mechanisms

**Figure 7:**
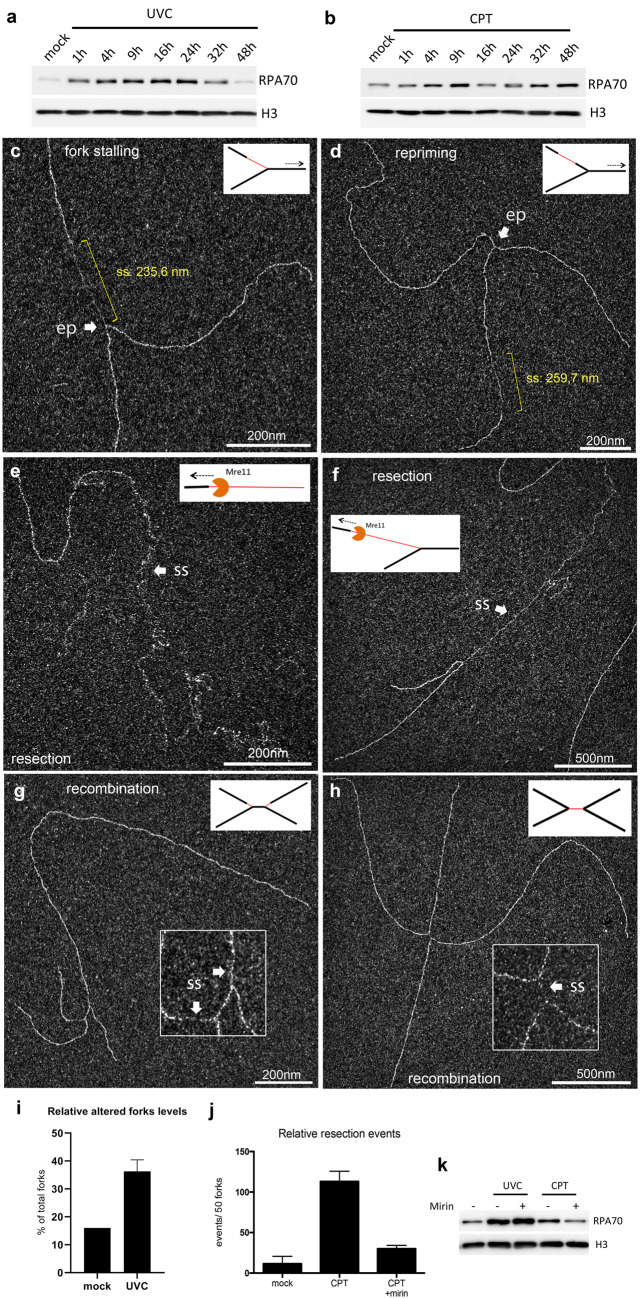
Characterization of *in vivo* DNA intermediates following genotoxic treatments reveals the cellular DDR. Kinetic characterization of global ssDNA accumulation by monitoring the RPA70 recruitment to chromatin after CPT (200 nM) (**a**) or UVC (15 J/m^2^) (**b**) treatment of human fibroblastic cells. UVC induced a one-way profile over the time, whereas CPT induced a two-way profile with ssDNA remaining unrepaired at 48 h. For TEM analysis, gDNA was isolated at 1 h after genotoxic treatment and subjected to BPS spreading. (**c**, **d**) Altered replication forks isolated from UV-treated cells (25 J/m^2^). Indicated in yellow, two types of ssDNA stretches could be observed and measured for further studies, one starting from the elongation point (**c**), reminiscent of a fork stalling, and the other located on one daughter strand (**d**), suggesting a postreplicative gap generated by a repriming process. (**e**, **f**) Early resection events isolated from CPT-treated cells (200 nM), consisting of long-ssDNA stretches (several kbs), indicated with arrows and located either at dsDNA molecule extremity (e) or at the elongation point of a replication fork (f). Multibranched structures (or “H-shape”) resulting from late recombination events in (**g**) UV-treated cells and (**h**) CPT-treated cells. (**i**) Relative altered forks levels upon UVC treatment. For each condition, 50 forks were characterized and evaluated for the UVC-specific ssDNA regions located at or/and behind the elongation point. The error bar represents the SD between two independent experiments. (**j**, **k**) MRC5 cells were pretreated or not with 30 µM Mirin for 1 h, and then subjected to CPT treatment for 1 h. (j) The levels of relative resection events were evaluated and normalized to 50 usual replication forks. The error bars represent the SD between two independent experiments. (k) Together with the levels of resection events, the addition of Mirin also reduced the global ssDNA levels following the CPT treatment, but not after UVC. ss, ssDNA; ep, elongation point.

The cellular response to replication stress is in part specific of the type of DNA lesion. To validate the whole procedure described herein, we characterized DNA intermediates structures in cells undergoing two different genotoxic treatments. On the one hand, UVC is natural solar radiations inducing bulky lesions into DNA, mostly composed of cyclobutane pyrimidine dimers and pyrimidine 6–4 pyrimidone (6–4 PP) photoproducts [72]. On the other hand, CPT is an anticancer cytotoxic alkaloid that stabilizes a cleavable complex between topoisomerase I and DNA. Both treatments impede the replication forks progression and generate genomic ssDNA accumulation [1, 73–78]. RPA is the major ssDNA-binding protein in eukaryotes and is a key factor in both DNA replication and DDR pathways activation [79, 80]. In order to evaluate the global genomic ssDNA accumulation upon both treatments, we monitored the recruitment of RPA70 subunit levels to the chromatin as a sensor of global DNA damages. Both UVC (15 J/m^2^) and CPT (200 nM) treatments induced a recruitment of RPA70 in the first hour, but the recruitment profiles evolved differently over the time (compare Fig. 7a and b).

To provide information on DDR mechanisms used by the cells to cope with the damages, DNA intermediates were characterized by TEM early (1 h) and later (2 h) after both equivalent treatments. In UVC-treated cells, we showed an early accumulation of ssDNA regions at and behind the elongation point of the replication forks (Fig. 7c and d, respectively). Previous TEM studies reported that it corresponded to the stalling of leading strand synthesis (inducing the uncoupling of helicase and synthesis activities) and to postreplicative gaps owing to repriming events [59–61]. Here the rate of these altered forks increased 2.3-fold upon UVC challenge (Fig. 7i). Conversely, CPT treatment induced the accumulation of much longer ssDNA regions located either at dsDNA molecule extremities or less frequently starting from the elongation point of the replication forks (Fig. 7e and f). The addition of Mirin, an Mre11 nuclease inhibitor, significantly reduced (almost 4-fold) the levels of these structures and limited RPA70 recruitment to chromatin (Fig. 7j and k). In contrast, the inhibitor did not influence RPA70 recruitment induced by UVC, revealing two distinct mechanisms for early ssDNA accumulation. These results were consistent with previous studies describing UV-induced fork stalling and gaps accumulation [59, 61], however, we provided here the first structural observations of early resection events induced by CPT treatment.

Two hours following both treatments, multibranched DNA intermediates were observed (Fig. 7g and h). Intriguingly, they were totally absent at earlier time points or in untreated conditions. Most of the time, they displayed an “H-shape” with a short ssDNA region at the center, making them unlikely to correspond to two converging replication forks. Interestingly, they locally resembled joint molecules identified in the *in vitro* strand-exchange reaction described above (Fig. 5h and j). We propose these structures to belong to postreplicative recombination events. However, further studies are needed to better characterize these particular DNA intermediates.

## Conclusion

TEM has a long history of success in characterizing the architecture of DNA intermediates through their direct visualization, which has undeniably provided a unique perspective for drawing the molecular basis of DNA replication and repair mechanisms. We present here methods specifically dedicated to structural analysis of replication and recombination DNA intermediates, including *in vitro* DNA and nucleoprotein machineries related to DNA repair pathways and *in vivo* replication DNA intermediates. It ranges from the preparation of *in vitro* and *in vivo* samples to imaging developments. The use of positive staining approach allows imaging in low-dye condition and will open perspectives to move toward a low-dose acquisition mode in order to improve resolution. These developments will be reinforced by the use of new generation of CCD or direct cameras associated with automatic acquisition system and adequate image analysis software. In this way, our team is actually developing the automatic digitization of DNA molecules by contour tracking, taking into account the different trajectories of the DNA.

Finally, while the method described herein represents an original and robust approach to characterize the molecular features of DDR pathways, we now have to explore new strategies to enrich and purify specific intermediate structures to enlarge and precise our application field. Coupled with other biochemical approaches, our method allowed dissecting-specific HR molecular mechanism, highlighting pioneering observations of resection and recombination events. Facing the structures observed *in vitro* and *in vivo* offers unique perspectives for the understanding of these complex molecular choreographies.

## Supplementary Material

bpaa012_Supplementary_Data
