# Supplementary material for: Method combining BAC film and positive staining for the characterization of DNA intermediates by dark-field electron microscopy
Source: Biol Methods Protoc. 2020 Jul 7;5(1):bpaa012. doi: 10.1093/biomethods/bpaa012 (PMC7474861; doi:10.1093/biomethods/bpaa012)
Supplement: bpaa012_Supplementary_Data [file bpaa012_supplementary_data.docx]

**Supplemental data**

**
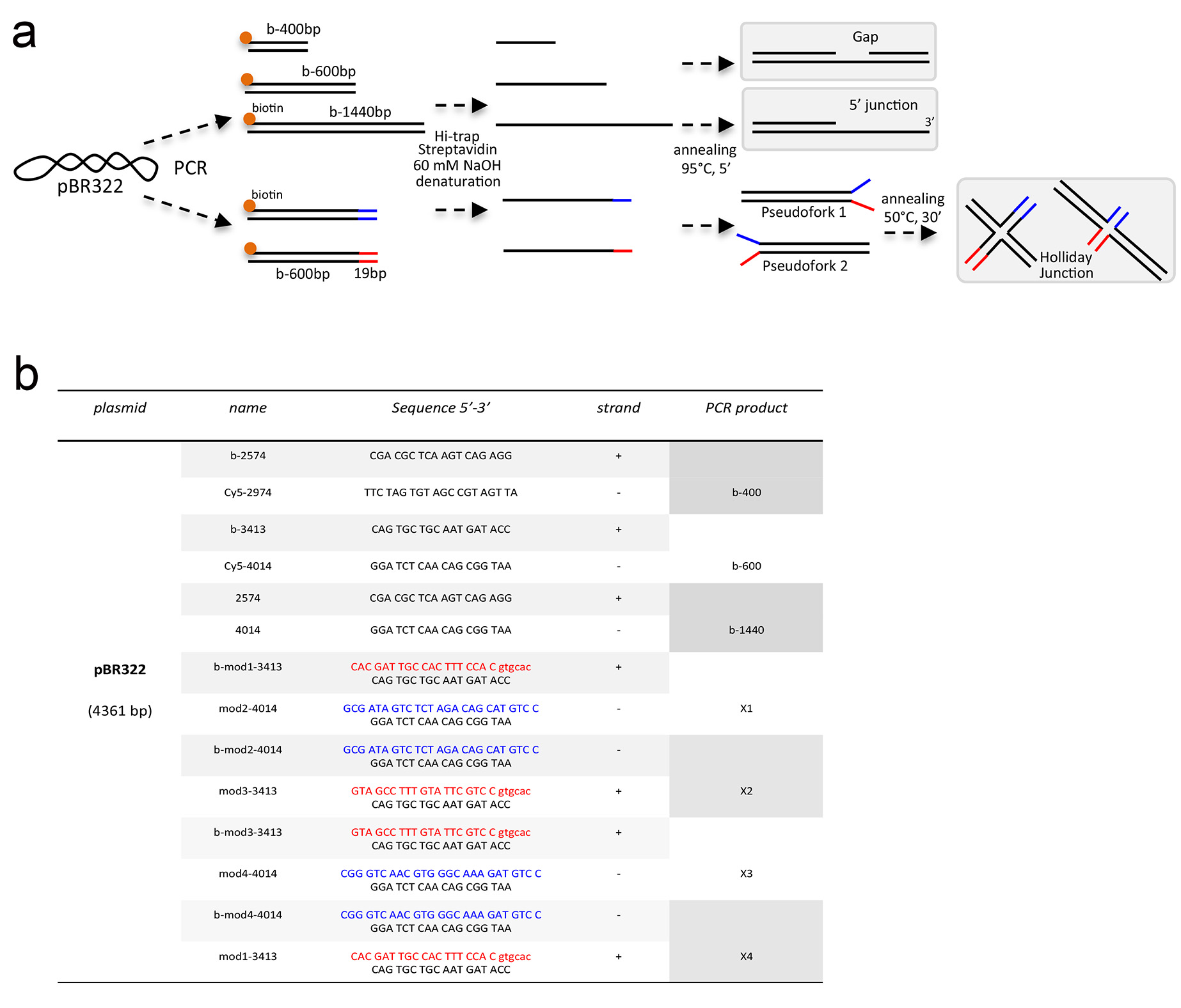
**

**Supplemental figure 1 Construction of DNA synthetic DNA substrates**

Scheme of the methodology implemented for synthetic gap, 5’ junction and Holliday Junction construction (a); table of the primers used to construct these substrates (b).
